# Critical COPD respiratory illness is linked to increased transcriptomic activity of neutrophil proteases genes

**DOI:** 10.1186/1756-0500-5-401

**Published:** 2012-08-02

**Authors:** Raquel Almansa, Lorenzo Socias, Monica Sanchez-Garcia, Ignacio Martín-Loeches, Milagros del Olmo, David Andaluz-Ojeda, Felipe Bobillo, Lucia Rico, Agueda Herrero, Vicente Roig, C Alicia San-Jose, Sara Rosich, Julia Barbado, Carlos Disdier, Raúl Ortiz de Lejarazu, Maria C Gallegos, Victoria Fernandez, Jesus F Bermejo-Martin

**Affiliations:** 1 Investigación Biomédica del Clínico (ibC), Hospital Clínico Universitario de Valladolid, Avda Ramón y Cajal 3, 47005, Valladolid, Spain; 2 Infection & Immunity Medical Investigation Unit (IMI), Microbiology and Immunology Service, Hospital Clínico Universitario - IECSCYL, Avda Ramón y Cajal 3, 47005, Valladolid, Spain; 3 Critical Care Department, Hospital Son Llatzer- SEMICYUC, Ctra. Manacor, km 4, 07198, Palma de Mallorca, Spain; 4 Respiratory Medicine Service, Hospital Clínico Universitario-SACYL / SEPAR /CIBERES, Avda Ramón y Cajal 3, 47005, Valladolid, Spain; 5 Critical Care Department Parc Tauli, Hospital Universitari de Sabadell Spain and CIBERES/ SEMICYUC, Sabadell, Spain; 6 Critical Care Department, Hospital Clínico Universitario-SACYL/SEMICYUC, Avda Ramón y Cajal 3, E-47005, Valladolid, Spain; 7 Critical Care Department, Hospital Universitari Joan XXIII, Mallafre Guasch 4, Tarragona, 43007, Spain; 8 Internal Medicine Service, Hospital Clínico Universitario de Valladolid - SACYL, Avda Ramón y Cajal 3, 47005, Valladolid, Spain; 9 Microbiology Service, Hospital Son Llatzer, Ctra. Manacor, km 4, 07198, Palma de Mallorca, Spain

**Keywords:** COPD, Critical, Expression, Gene, Microarray, Neutrophil, Proteases

## Abstract

**Background:**

Gene expression profiling (GEP) in cells obtained from peripheral blood has shown that this is a very useful approach for biomarker discovery and for studying molecular pathogenesis of prevalent diseases. While there is limited literature available on gene expression markers associated with Chronic Obstructive Pulmonary Disease (COPD), the transcriptomic picture associated with critical respiratory illness in this disease is not known at the present moment.

**Findings:**

By using Agilent microarray chips**,** we have profiled gene expression signatures in the whole blood of 28 COPD patients hospitalized with different degrees of respiratory compromise.12 of them needed of admission to the ICU, whilst 16 were admitted to the Respiratory Medicine Service. GeneSpring GX 11.0 software was used for performing statistical comparisons of transcript levels between ICU and non-ICU patients. Ingenuity pathway analysis 8.5 (IPA) and the Kyoto Encyclopedia of Genes and Genomes (KEGG) were used to select, annotate and visualize genes by function and pathway (gene ontology). T-test showed evidence of 1501 genes differentially expressed between ICU and non-ICU patients. IPA and KEGG analysis of the most representative biological functions revealed that ICU patients had increased levels of neutrophil gene transcripts, being [cathepsin G (CTSG)], [elastase, neutrophil expressed (ELANE)], [proteinase 3 (PRTN3)], [myeloperoxidase (MPO)], [cathepsin D (CTSD)], [defensin, alpha 3, neutrophil-specific (DEFA3)], azurocidin 1 (AZU1)], and [bactericidal/permeability-increasing protein (BPI)] the most representative ones. Proteins codified by these genes form part of the azurophilic granules of neutrophils and are involved in both antimicrobial defence and tissue damage. This “neutrophil signature” was paralleled by the necessity of advanced respiratory and vital support, and the presence of bacterial infection.

**Conclusion:**

Study of transcriptomic signatures in blood suggests an essential role of neutrophil proteases in COPD patients with critical respiratory illness. Measurement and modulation of the expression of these genes could present an option for clinical monitoring and treatment of severe COPD exacerbations.

## Findings

### Background

Patients with Chronic Obstructive Pulmonary Disease (COPD) suffer from periodical exacerbations, characterized by recurrent episodes of worsening respiratory symptoms. Exacerbations result in further decreases in lung function, impairing the patient’s quality of life, and increasing the use of healthcare resources [1]. In addition, development of Community Acquired Pneumonia (CAP) is also common in COPD exacerbations [2]. Gene expression profiling (GEP) in cells obtained from peripheral blood has proved to be a very useful non-invasive approach for biomarker discovery and for studying molecular pathogenesis of prevalent diseases [3]. While there is previous work identifying gene expression markers associated with COPD using Peripheral Blood Mononuclear Cells (PBMCs) [4], the transcriptomic picture associated with critical respiratory illness in this disease is not known at the present moment. The objective was to describe the gene expression signatures associated with critical COPD respiratory illness compared to non-critical COPD exacerbations, as a preliminary study aimed to identify the genes associated with the severity of this disease.

## Materials and methods

### Patients and samples

12 patients with pre-existing diagnosis of COPD in need of admission to the Intensive Care Unit due to acute respiratory failures were compared to a group of 16 COPD patients with non critical disease exacerbation admitted to the Respiratory Medicine Service. 4 healthy voluntary donors of similar ages to the patients were recruited for gene expression data normalization. A sample of 2.5 ml of blood was collected in the first 24 hours following admission to the ICU or to the Respiratory Medicine Service. Acute exacerbation of COPD was defined as a patient showing 2 symptoms (at least 1 major) for 2 consecutive days. Major symptoms were defined as increased dyspnoea, sputum volume, or sputum purulence and minor symptoms were increased cough, wheeze, sore throat, or coryzal symptoms. Definition of CAP was based on current American Thoracic Society and Infectious Disease Society of America guidelines [5] . Informed consent was obtained directly from each patient before enrolment. Patient’s identification remained anonymous. The protocol was approved by the Ethics Committee on Clinical Research of each one of the participating centers.

### Participant institutions

1. Investigación Biomédica del Clínico (ibC), Hospital Clínico Universitario de Valladolid, Avda Ramón y Cajal 3, 47005 Valladolid, Spain

2. Infection & Immunity Medical Investigation Unit (IMI), Microbiology and Immunology Service, Hospital Clínico Universitario, Valladolid, Spain - IECSCYL, 3. Critical Care Department, Hospital Son Llatzer, Palma de Mallorca, Spain - SEMICYUC 4. Respiratory Medicine Service, Hospital Clínico Universitario-SACYL / SEPAR, Valladolid, Spain 5. Critical Care Department. Parc Tauli, Hospital Universitari de Sabadell, Spain. 6. Critical Care Department, Hospital Clínico Universitario-SACYL/SEMICYUC, Valladolid, Spain. 7. Critical Care Department, Hospital Universitari Joan XXIII. Tarragona, Spain. 8. Internal Medicine Service. Hospital Clínico Universitario - SACYL, Valladolid, Spain. 9. Microbiology Service, Hospital Son Llatzer, Palma de Mallorca, Spain.

### Microbial diagnosis

Sputum samples were routinely Gram stained and cultured on general purpose media (blood agar, chocolate agar, and the differentials mediums McConkey agar and Chapman agar). Fungal infections were screened by culturing sputum samples on Sabouraud agar containing chloramphenicol [6]. Viral diagnosis was performed on RNA from pharyngeal swabs collected in the first 24 hours following admission to the hospital by reverse transcription-polymerase chain reaction–based methods using reagents purchased from Roche™ (Swine Inf A/H1N1 detection set). These samples were also assessed by multiplex polymerase chain reaction (Luminex) with xTAG RVP kit from Luminex-Abbott for infection with Respiratory Syncytial Virus, Influenza B virus, Parainfluenza viruses 1–4, Human metapneumovirus, Enteroviruses, Rhinovirus, Adenovirus, Bocavirus and Coronaviruses NL63, HKU1, 229E and OC43, in accordance with manufacturer’s instructions.

### Microarray processing

Total RNA was extracted from blood samples using the PAXgene Blood RNA System (PreAnalytix, Hombrechtikon, Switzerland). RNA was quantified by spectrometry (NanoDrop ND1000, NanoDrop Technologies, Wilminton, Delaware USA) and quality confirmed by RNA Experion Bioanalyzer (BioRad, California USA) assay. Up to 1750 ng of each RNA sample was concentrated with the RNeasy MinElute Cleanup kit (QIAGEN, Hilden, Germany). RNA was eluted with 10 microliter of RNase-free H2O. 300 ng of purified total RNA were used to produce Cyanine 3-CTP-labeled cRNA using the Quick Amp Labeling kit (Agilent p/n 5190–0442) according to the manufacturer’s instructions. Following ‘One-Color Microarray-Based Gene Expression Analysis’ protocol Version 5.7 (Agilent p/n 4140–90040), 3 μg of labeled cRNA was hybridized with Whole Human Genome Oligo Microarray Kit (Agilent p/n G2519F-014850) containing 41,000+ unique human genes and transcripts. Arrays were scanned in an Agilent Microarray Scanner (Agilent G2565BA) according to the manufacturer’s protocol and data extracted using Agilent Feature Extraction Software 9.5.3 following the Agilent protocol GE1-v5_95_Feb07 and the QC Metric Set GE1_QCMT_Jan08. Resulting microarray data sets have been uploaded to the ArrayExpress microarray data repository [accession number: E-MEXP-3589]. Changes were verified in microarray gene expression for representative genes of our analysis by qPCR using Real-time Ready plates purchased to Roche, using β-actin and β2-microglobulin as reference (housekeeping) genes.

### Data analysis

Data analysis was carried out by using GeneSpring GX 11.0 software. The original data was cleansed and normalized using the robust multichip average (RMA) algorithm consists of three steps: background correction, p75 normalization and expression calculation. Subsequent to logarithms transformation, baseline transformation of the data was performed using the median of control samples. Before statistical analyses all microarrays were subjected to quality and filtering criteria. Quality of the microarray data was assessed on Principal Component analysis (PCA) plots. All the 32 arrays passed these criteria and were included in the analyses. Student T tests (GeneSpring GX11.0) were used to identify genes differentially expressed between ICU and non-ICU groups at the level of significance p < 0.01 with Benjamini-Hochberg multiple testing corrections. GeneSpring GX 11.0 was used also for performing gene hierarchical clustering. Ingenuity pathway analysis 8.5 (IPA) (Ingenuity Systems, Redwood City, CA) and the Kyoto Encyclopedia of Genes and Genomes (KEGG) were used to select, annotate and visualize genes by function and pathway (gene ontology).

## Results

**Clinical description of the patients (Table**1): non-critically ill patients were slightly older than critically ill ones (p < 0.1). Male patients accounted for the vast majority of the cases in the non-ICU group. Sex distribution was more equal in the group of critically ill patients. CAP at admission was more frequent in the ICU group. No differences in the frequency of viral infection were found between groups. On the contrary, bacterial or fungal infection was more frequent in the ICU group. While both groups showed severe hypoxemia at admission, critically ill patients showed higher levels of CO_2_ in their blood. Five critical patients needed invasive mechanical ventilation. None of the critically ill patients showed higher lymphocyte counts in their blood. On the contrary, critically ill patients showed higher neutrophil counts. None of the patients died. Microbiological results revealed the following distribution of viral and bacterial infection: **Respiratory virus infection**: Non ICU group: Rhinovirus (n = 4); Influenza A/H1N1 nv (n = 1); Metapneumovirus (MPV) (n = 1); Coronavirus 229E (n = 1). ICU group: MPV (n = 1); Influenza A/H1N1 nv (n = 2); Respiratory Syncytial Virus (RSV) (n = 1). **Bacterial/fungal infection**: Non ICU group: P aeruginosa (n = 2); Candida glabrata (n = 1). ICU group: A. fumigatus (n = 1); C. pneumoniae (n = 1); S. pneumoniae (n = 3); S hominis (n = 1); P aeruginosa (n = 1); S. epidermidis (n = 1). Co-infection (viral + bacterial infection): non ICU group: [Rhinovirus + P aeruginosa] (n = 2). ICU group: the following co-infections were found: [Influenza A/H1N1 nv + A. fumigatus] (n = 1); [RSV + S pneumoniae] (n = 1); [MPV + S. epidermidis] (n = 1). All patients were treated with bronchodilators, antibiotics, and systemic steroids (methylprednisolone i.v., 20–40 mg/day). Treatment decisions for all patients were not standardized and were decided by the attending physician.

**Table 1:** Clinical characteristics of the patients

	**No ICU (n = 16)**	**ICU (n = 12)**	***p***
**Age (Mean,SD)**	73.4	65.6	0.094
	(9.0)	(11.4)	
**Sex (M/F)**	15/1	7/5	0.024
**Community Acquired Pneumonia (YES / NO)**	3/13	8/4	0.005
**Respiratory Virus (YES / NO)**	7/9	4/8	n.s
**Bacterial / fungal infection (YES / NO)**	3/13	8/4	0.027
**PaO2 at admisión (Mean,SD)**	58.7	61.9	n.s
	(13.5)	(22.7)	
**PCO2 at admisión (Mean,SD)**	43.3	63.1	0.050
	(14.2)	(36.7)	
**Invasive Mechanical Ventilation (YES / NO)**	0/16	5/7	0.004
**Lymphocytes / mm**^**3**^** in blood (Mean,SD)**	8249.7	964.1	0.000
	(6108.6)	(437.9)	
**Monocytes / mm**^**3**^** in blood (Mean,SD)**	2265.3	596.1	0.064
	(3647.2)	(622.5)	
**Neutrophils / mm**^**3**^** in blood (Mean,SD)**	1255.0	13301.2	0.000
	(1845.8)	(11858.8)	

Mann Whitney U test and Chi-squared test were employed to compare mean and proportions between groups were appropriated.

### Gene expression analysis

T-test evidenced 1501 genes differentially expressed between ICU and non ICU patients. ICU patients showed higher expression levels of 558 genes and lower expression levels of 943 than non critically ill patients.

### Genes showing higher levels of RNAm transcripts in the ICU group

**Figure 1:**
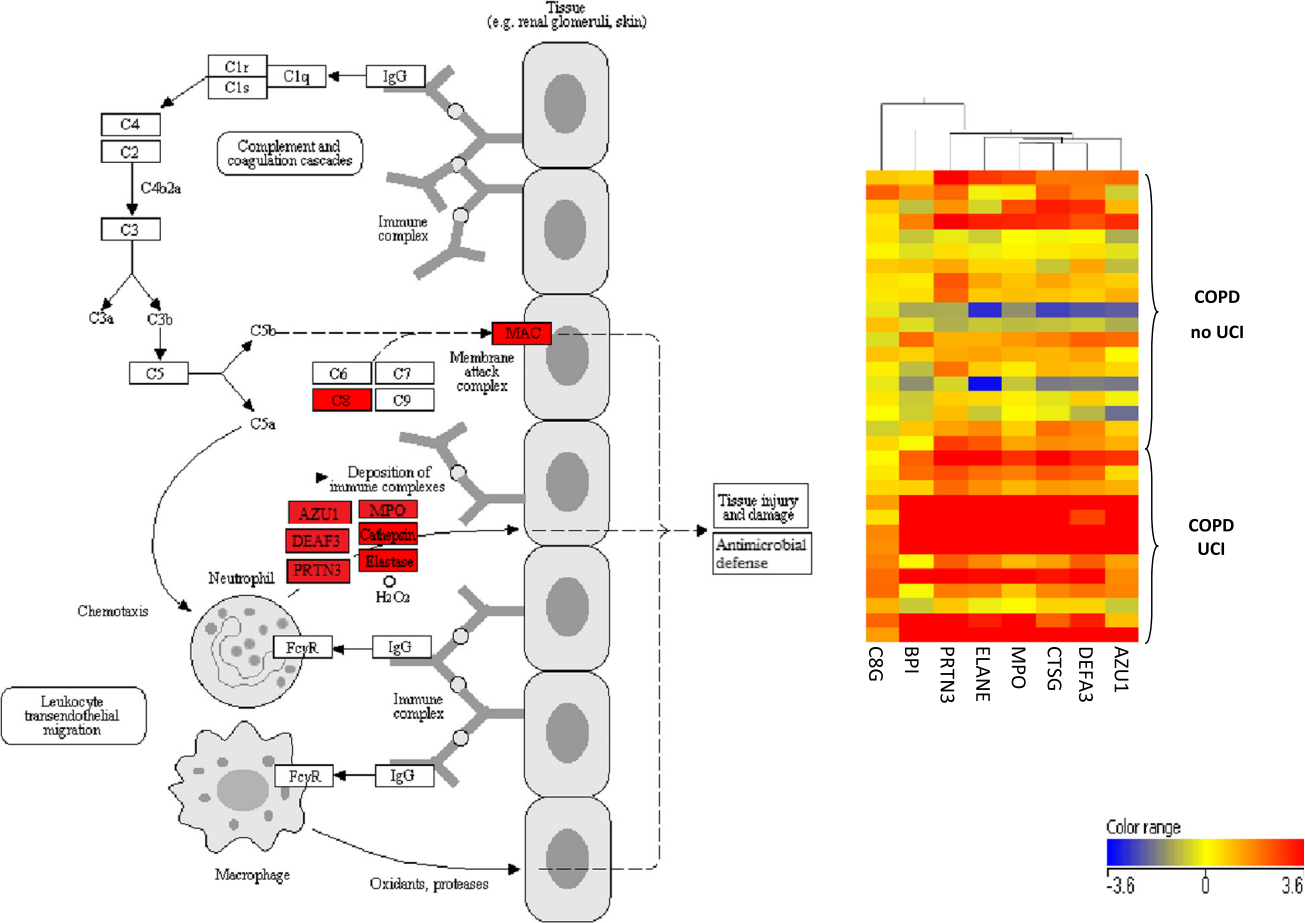
**(a) Left: model depicting the physiological roles of neutrophil and complement system.** Those genes with relative higher expression in the ICU compared to the no ICU group are depicted in red (modified from KEGG) (**b**) One-way hierarchical clustering of neutrophil protease genes. Signal intensity data are color-coded such that the intensity of red indicates a relatively high level of expression, while the intensity of blue represents a relatively low level of expression.

IPA analysis of the most representative biological functions revealed that those genes showing higher levels of their corresponding transcripts in the ICU group participate principally in four biological functions: [immune response] (Table 2), [severe acute respiratory syndrome], [respiratory infection] (Table 3) and [inflammatory response] (Table 4). Patients in the ICU group not only showed increased neutrophil counts in blood, but also higher levels of RNAm corresponding to a group of proteins known to participate in neutrophil-mediated antimicrobial defense and tissue injury (Figure 1).

**Table 2:** Gene expression levels by biological function of genes relatively up-regulated in the ICU group, based upon IPA results

**Immune Response**					
**IPA p-Value**					
**3,38E-08**					
**GeneSymbol**	**Corrected p-value**	**FC Absolute**	**GeneSymbol**	**Corrected p-value**	**FC Absolute**
**LCN2**	0,004	10,37	**MAGEA3**	0,028	3,13
**LTF**	0,004	9,86	**LAIR1**	0,013	2,99
**RETN**	0,014	8,85	**FCAR**	0,024	2,98
**ELANE**	0,026	8,19	**PAX6**	0,041	2,92
**BPI**	0,018	7,06	**APCS**	0,046	2,88
**INHBA**	0,007	7,12	**MEFV**	0,020	2,83
**CHIT1**	0,009	4,19	**CTSD**	0,037	2,82
**HP**	0,015	6,71	**PYDC1**	0,013	2,77
**PRTN3**	0,030	6,33	**GNAO1**	0,032	2,67
**AZU1**	0,038	6,13	**ELN**	0,037	2,63
**CTSG**	0,034	5,71	**MGLL**	0,023	2,61
**MPO**	0,024	4,97	**VEGFA**	0,019	2,60
**CEACAM8**	0,027	4,80	**AKT2**	0,007	2,49
**PGLYRP1**	0,016	4,54	**GRK6**	0,014	2,39
**CEBPE**	0,009	4,49	**MAP2K3**	0,045	2,37
**GAST**	0,020	4,09	**TIMP1**	0,009	2,33
**PGF**	0,039	4,07	**ABCA7**	0,007	2,32
**APP**	0,004	4,06	**ITGAX**	0,021	2,26
**RNASE2**	0,007	3,69	**POMC**	0,038	2,25
**DEFA3**	0,024	3,68	**S100A12**	0,031	3,40
**BLOC1S3**	0,009	3,68	**TRPC2**	0,034	3,37
**MPP1**	0,025	3,61	**CAPG**	0,031	2,08
**CD24**	0,029	3,54	**KSR1**	0,040	3,19
**RAP1GAP**	0,034	3,46	**GBA**	0,007	3,14
**STK11**	0,020	2,23	**MUC1**	0,030	2,06
**GFI1B**	0,045	2,17	**GABARAPL2**	0,028	2,04
**BAD**	0,007	2,17	**C8G**	0,017	2,03
**CYBA**	0,011	2,16	**UNC13D**	0,014	2,02
**PRDX5**	0,010	2,11	**SBDS**	0,033	2,01

**FC:** fold change of gene expression values in the ICU / non ICU group.

**Table 3:** Gene expression levels by biological function of genes relatively up-regulated in the ICU group, based upon IPA results

**Severe acute respiratory syndrome & Infection of respiratory tract**		
**IPA p-Value 5,66E-19 and 7,46E-18 respectively**		
**GeneSymbol**	**Corrected p-value**	**FCAbsolute**
**LCN2**	0,004	10,37
**LTF**	0,004	9,86
**BPI**	0,012	7,20
**HP**	0,015	6,71
**CEACAM8**	0,027	4,80
**PGLYRP1**	0,016	4,54
**CEBPE**	0,009	4,49
**TCN1**	0,017	3,86
**RNASE2**	0,007	3,69
**DEFA3**	0,024	3,68
**CD24**	0,029	3,54
**S100A12**	0,031	3,40
**HIST1H2AC**	0,011	3,04
**HIST1H1C**	0,010	2,90
**APCS**	0,046	2,88
**RAB13**	0,019	2,63
**STXBP2**	0,034	2,55
**GAPDH**	0,039	2,52
**TIMP1**	0,009	2,33
**PDXK**	0,015	2,26
**TUBB2C**	0,010	2,16

**FC:** fold change of gene expression values in the ICU / non ICU group.

**Table 4:** Gene expression levels by biological function of genes relatively up-regulated in the ICU group, based upon IPA results

**Inflammatory response**		
**IPA p-Value: 5,70E-07**		
**GeneSymbol**	**Corrected p-value**	**FCAbsolute**
**LCN2**	0,004	10,37
**LTF**	0,004	9,86
**ELANE**	0,026	8,19
**BPI**	0,018	7,06
**INHBA**	0,007	7,12
**HP**	0,015	6,71
**PRTN3**	0,030	6,33
**AZU1**	0,038	6,13
**CTSG**	0,034	5,71
**MPO**	0,024	4,97
**CEBPE**	0,009	4,49
**APP**	0,004	4,06
**RNASE2**	0,007	3,69
**DEFA3**	0,024	3,68
**MPP1**	0,025	3,61
**RAP1GAP**	0,034	3,46
**FCAR**	0,024	2,98
**APCS**	0,046	2,88
**MEFV**	0,020	2,83
**GNAO1**	0,032	2,67
**ELN**	0,037	2,63
**MGLL**	0,023	2,61
**VEGFA**	0,019	2,60
**GRK6**	0,014	2,39
**MAP2K3**	0,045	2,37
**POMC**	0,038	2,25
**CYBA**	0,011	2,16
**PRDX5**	0,010	2,11
**MUC1**	0,030	2,06
**UNC13D**	0,014	2,02
**SBDS**	0,033	2,01

**FC:** fold change of gene expression values in the ICU / non ICU group.

### Genes showing lower levels of RNAm transcripts in the ICU group

IPA analysis revealed that those genes showing lower levels of their corresponding transcripts in the ICU group participate in the immune response against pathogens: [Natural Killer Cell Signaling], [T Cell Receptor Signaling], [Regulation of IL-2 Expression in Activated and Anergic T Lymphocytes], [Transforming Growth FActor-β Signaling], [CCR5 Signaling in Macrophages], [CD28 Signaling in T Helper Cells] (Additional file 1: Table S1 and Additional file 2: Table S2).

### Real time qPCR results

Differential expression was confirmed in samples from all patients by qPCR. Differences between gene expression levels in the qPCR assays were assessed by performing the Mann–Whitney U test and by using SPSS 20.0 software analysis (significance was fixed at the level *p* < 0.05), as shown in Additional file 3: Figure S1.

## Discussion

In our study, a comprehensive GEP study was performed on whole blood, which revealed the existence of increased expression levels of a group of neutrophil genes associated with antimicrobial defence and tissue damage, as molecular signature of critical respiratory illness in COPD patients.

### Genes showing increased expression in the ICU group

#### Neutrophil related genes

ELANE (Elastase, neutrophil expressed), CTSG (cathepsin G), and PRTN3 (proteinase 3) are neutrophil serine proteases (NSPs) produced during neutrophil development in bone marrow and stored in the azurophilic granules of mature neutrophils. NSPs contribute to the neutrophil oxygen-independent system-mediated protection of the host against invading pathogens [7]. On the other hand, NSPs play a critical role in neutrophil-associated lung inflammatory and tissue-destructive diseases, including COPD. NSPs have a broad substrate specificity and degrade a variety of extra-cellular matrix proteins including elastin, collagen (type I–IV), fibronectin, laminin and proteoglycans [8]. High concentrations of NSPs are found in purulent secretions of COPD patients[9]. Intra-tracheal instillation of mice with human neutrophil elastase or proteinase 3 leads to tissue destruction and airspace enlargement [10]. Neutrophil elastase may also impair host defence interfering with muco-ciliary clearance of bacteria and phagocytosis of pathogens. In turn, both elastase and cathepsin G impair T-cell function through cleavage of CD2, CD4 and CD8 on the surface of T-cells [8]. Patients lacking α1-Pi, the main physiological inhibitor of neutrophil elastase, are at greater risk of developing emphysema [11]. CTSD (Cathepsin D) is also present in azurophil granules. By using a mice model, Bracke et al.. have recently demonstrated that cigarette smoke induces the expression of CTSD in pulmonary macrophages [12], supporting the role of cathepsins in the respiratory compromise associated to COPD . MPO (Myeloperoxidase) constitutes the major component of neutrophil azurophilic granules. MPO is important in bacterial killing, but also drives inflammatory reactions and tissue oxidation. By using a mice model of influenza infection, Sugamata R et al.. found that the absence of MPO reduced inflammatory damage with suppression of leakage of total proteins in bronchoalveolar lavage fluid associated with alteration of claudins in the lung [13]. Activated neutrophils (MPO + cells) are found in severe COPD [14]. In this sense, inhibition of MPO may be a novel and useful therapeutic treatment for COPD [15]. In turn, AZU1 (azurocidin 1), BPI (bactericidal/permeability-increasing protein) and alpha-defensins (such as DEFA3, defensin, alpha 3, neutrophil-specific), are as NSPs, major constituents of neutrophil azurophilic granules. The protein encoded by AZU1 is an antibiotic protein, with monocyte chemotactic and antibacterial activity. It is also an important multifunctional inflammatory mediator. Bactericidal/permeability-increasing protein (BPI) encodes a lipopolysaccharide binding protein with bactericidal activity on gram-negative organisms. Defensins show activities against Gram-positive and Gram-negative bacteria, fungi, yeast, and enveloped viruses, but, as NSPs, also play important roles in promoting inflammation in the lungs, potentially contributing to lung injury [16] . Along with NSPs, defensins may also be released upon neutrophil stimulation. Both have been described to affect the integrity of the epithelial layer, decrease the frequency of ciliary beat, increase the secretion of mucus, and induce the synthesis of epithelium-derived mediators that may influence the amplification and resolution of neutrophil-dominated inflammation [8].

A number of other gene transcripts related to neutrophils were identified in our analysis as more represented in the ICU group: LTF (lactotransferrin), TCN1 (transcobalamin I (vitamin B12 binding protein, R binder family)) as proteins which form part of secondary granules in neutrophils. S100A12 (S100 calcium binding protein A12) which codifies a protein proposed to be involved in specific calcium-dependent signal transduction pathways and which regulatory effect on cytoskeletal components may modulate various neutrophil activities. STXBP2 (Syntaxin binding protein 2) is involved in neutrophil degranulation. MPP1 (Membrane protein, palmitoylated 1, 55 kDa), which participates in the regulation of neutrophil chemotaxis. FCAR (Fc fragment of IgA, receptor for): this protein interacts with IgA-opsonized targets and triggers several immunologic defense processes, including phagocytosis. ITGAX (Homo sapiens cDNA, FLJ99683) which mediates adherence of neutrophils and monocytes to stimulated endothelium cells, and in the phagocytosis of complement coated particles. FCAR: this gene encodes a receptor for the Fc region of IgA, present on the surface of myeloid lineage cells such as neutrophils, monocytes, macrophages, and eosinophils, and triggers several immunologic defence processes, including phagocytosis, antibody-dependent cell-mediated cytotoxicity, and stimulation of the release of inflammatory mediators.

This “neutrophil signature” supports the notion of the existence at the systemic level of neutrophils ready to fight against infection but also ready to produce mediators which are able to induce tissue injury. Participation of these molecules in COPD pathogenesis had already been described at the respiratory level, but this work is the first suggesting an important activity of these enzymes at the systemic level. Migration of these cells from the blood vessels could certainly help to clear the causative microbe, but also to damage the pulmonary parenchyma, contributing to the explanation of the severity of respiratory conditions of these patients. Finding this “neutrophil signature” was possible since we used whole blood instead of PBMCs for the transcriptomic study [4]. This signature is probably related to the fact that the vast majority of patients in the ICU group showed bacterial infection, and neutrophils are major actors in antibacterial defence. In fact, critically ill patients in our cohort showed significantly higher total counts of neutrophils in blood than non-critically ill ones. On the other hand, a limitation of our work is that we did not isolated neutrophils for the gene expression profiling assays. Further works targeting specific cell types would contribute to the definition of the exact contribution of neutrophils and other leukocytes to the gene expression signatures linked to severe respiratory illness in patients with COPD.

#### Other genes showing increased expression in the ICU group

C8G (Complement component 8, gamma polypeptide) forms part of C8, protein which plays a central role in assembly of the "membrane attack complex" (MAC) of complement. The MAC is a macromolecular pore that targets and lyses pathogens that challenge the host. Deposition of these pores on human cells contributes to tissue damage. Interestingly, MPO products seem to be able to activate complement [17], indicating a potential link between MPO over-expression and induction of MAC formation. CD24: This gene encodes a sialoglycoprotein that is expressed on mature granulocytes and in many B cells. Other interesting gene relatively up-regulated in critical patients was VEGFA (Vascular endothelial growth factor A). This protein is a glycosylated mitogen that specifically acts on endothelial cells and has various effects, including mediating increased vascular permeability, inducing angiogenesis, vasculogenesis and endothelial cell growth, promoting cell migration, and inhibiting apoptosis. Circulating levels of VEGF are up-regulated in patients with acutely exacerbated COPD and decrease after recovery from exacerbation [18]. Respiratory levels of VEGF have been shown to negatively correlate with pulmonary function in stable COPD, which suggests its important role in COPD airway remodelling [19]. ELN (Elastin): This gene encodes a protein that is one of the two components of elastic fibbers, and participates in extracellular matrix organization, such as those taking place after severe tissue injury. MUC1 (Mucin 1, cell surface associated) play an essential role in forming protective mucous barriers on epithelial surfaces and in the response to hypoxia. Finally, we identified increased transcript levels in the ICU group of a group of genes involved in antibacterial defence: PGLYRP1 (peptidoglycan recognition protein 1), CEBPE (CCAAT/enhancer binding protein (C/EBP), epsilon), CHIT1 (chitinase 1 (chitotriosidase)) and CYBA (cytochrome b-245, alpha polypeptide).

### Genes showing decreased expression in the ICU group

On the other hand, the significantly lower counts of lymphocytes and monocytes in the blood of critically ill patients compared to non-critically ill ones probably explains the relatively depressed expression of immune related genes found in the former group. Migration of these cells to the site of infection (lung) or increased apoptosis in this group of patients could contribute to the observed lymphopenia and monocytopenia [3]. Alternatively, the presence of an increased nitro-oxidative stress environment and the critical condition of the most severe patients could down-modulate the expression of genes involved in the response against pathogens.

## Conclusion

Study of transcriptomic signatures in blood suggests the existence of an intense activity of neutrophil proteases in COPD patients with critical respiratory illness due to a severe exacerbation. These enzymes are involved in antimicrobial defense but could also mediate tissue damage in the lungs, and as a consequence this would explain the severe respiratory failure observed in these patients. Further studies will determine whether measurement / modulation of the expression of these genes could represent an option for clinical monitoring and treatment of severe COPD exacerbations.

### Availability of supporting data

The data sets supporting the results of this article are available in the ArrayExpress repository; reference number E-MEXP-3589.

## Abbreviations

COPD: Chronic obstructive pulmonary disease; IPA: Ingenuity pathway analysis; KEGG: Kyoto encyclopedia of genes and genomes; CTSG: Cathepsin G; ELANE: Elastase, neutrophil expressed; PRTN3: Proteinase 3; MPO: Myeloperoxidase; CTSD: Cathepsin D; DEFA3: Defensin, alpha 3, neutrophil-specific; AZU1: Azurocidin 1; BPI: Bactericidal/permeability-increasing protein; NSPs:Neutrophil serine proteases.

## Competing interests

The authors declare that they have no competing interests

## Authors’ contributions

LS, MSG, MDO, DAO, FB, AH, SR, VR, JB participated in patient recruitment and assisted in the analysis, interpretation of data, and writing the report. RA developed the bioinformatics analysis. LR performed the gene expression profiling works. IML and JFBM assisted in the design of the study, coordinated patient recruitment, analyzed and interpreted the data, and assisted in writing the paper. CASAJ introduced the clinical data and helped with data interpretation. MCG, VF performed the microbiology diagnosis and assisted in writing the paper. ROL and CD assisted in writing the paper. All authors read and approved the final manuscript.

## Supplementary Material

Additional file 1 **Table S1. Gene expression levels by intracellular signalling pathway of genes relatively down-regulated in the ICU group, based upon IPA results.** FC: fold change of gene expression values in the ICU / non ICU group. Click here for file

Additional file 2 **Table S2. Gene expression levels by intracellular signalling pathway of genes relatively down-regulated in the ICU group, based upon IPA results.** FC: fold change of gene expression values in the ICU / non ICU group. Click here for file

Additional file 3 **Figure S1. Gene expression levels by Quantitative Real time PCR.** Crossing points (CP) were calculated for the target gene and for the house-keeping genes (β -actin and β2-microglobulin). The ratio between the CP for the target gene / mean of the CPs for the housekeeping genes was calculated, and the inverse of this ratio is represented in each group of patients (ICU and no ICU). This way, the higher the median in the box-plot, the higher is the expression of the target gene. Differences were significant at the level *p* < 0.05. Click here for file
