# Supplementary material for: Critical COPD respiratory illness is linked to increased transcriptomic activity of neutrophil proteases genes
Source: BMC Res Notes. 2012 Aug 2;5:401. doi: 10.1186/1756-0500-5-401 (PMC3475085; doi:10.1186/1756-0500-5-401)
Supplement: Additional file 1 — Table S1. Gene expression levels by intracellular signalling pathway of genes relatively down-regulated in the ICU group, based upon IPA results. FC: fold change of gene expression values in the ICU / non ICU group. [file 1756-0500-5-401-S1.doc]

|  | **GeneSymbol** | **Fold change** |
| --- | --- | --- |
| **NK cell**  **Signalling**  IPA  (-log p value 6.69 E00) | AKT3 | -2.566 |
|  | CD247 | -3.533 |
|  | KIR2DL2 | -2.374 |
|  | KLRA1 | -2.532 |
|  | KLRB1 | -2.445 |
|  | KLRC1 | -3.342 |
|  | KLRC3 | -3.465 |
|  | KLRD1 | -4.037 |
|  | KLRK1 | -3.465 |
|  | PIK3C2A | -2.488 |
|  | PIK3C2B | -2.082 |
|  | PRKCH | -3.091 |
|  | PRKD3 | -2.153 |
|  | RRAS2 | -4.116 |
|  | SH2D1A | -4.359 |
|  | SH2D1B | -4.168 |
|  | VAV2 | -2.568 |
| **T cell**  **Receptor**  **Signalling**  IPA  (-log p value 6.38E00) | BCL10 | -3.117 |
|  | CD28 | -3.620 |
|  | CD247 | -3.533 |
|  | CD3E | -2.909 |
|  | CD8A | -3.531 |
|  | CTLA4 | -2.336 |
|  | ITK | -3.185 |
|  | MALT1 | -2.134 |
|  | MAPK8 | -2.126 |
|  | PIK3C2A | -2.488 |
|  | PIK3C2B | -2.082 |
|  | PPP3CC | -2.112 |
|  | RASGRP1 | -2.914 |
|  | RRAS2 | -4.116 |
|  | TXK | -2.395 |
|  | VAV2 | -2.568 |
| **Regulation of**  **IL-2**  **Expression**  **in Activated**  **and**  **Anergic T**  **Lymphocytes**  IPA  (-log p value 3.24E00) | BCL10 | -3.117 |
|  | CD28 | -3.620 |
|  | CD247 | -3.533 |
|  | CD3E | -2.909 |
|  | MALT1 | -2.134 |
|  | MAPK8 | -2.126 |
|  | PPP3CC | -2.112 |
|  | RRAS2 | -4.116 |
|  | SMAD3 | -2.360 |
|  | VAV2 | -2.568 |
