# Supplementary material for: Critical COPD respiratory illness is linked to increased transcriptomic activity of neutrophil proteases genes
Source: BMC Res Notes. 2012 Aug 2;5:401. doi: 10.1186/1756-0500-5-401 (PMC3475085; doi:10.1186/1756-0500-5-401)
Supplement: Additional file 2 — Table S2. Gene expression levels by intracellular signalling pathway of genes relatively down-regulated in the ICU group, based upon IPA results. FC: fold change of gene expression values in the ICU / non ICU group. [file 1756-0500-5-401-S2.doc]

|  | **GeneSymbol** | **Fold change** |
| --- | --- | --- |
| **TGF-β Signaling**  IPA  (-log p value 3.07E00) | ACVR2B | -3.743 |
|  | BCL2 | -2.456 |
|  | BMPR2 | -2.451 |
|  | BMPR1A | -2.124 |
|  | MAPK8 | -2.126 |
|  | PIAS4 | -3.391 |
|  | RRAS2 | -4.116 |
|  | RUNX3 | -2.844 |
|  | SMAD3 | -2.360 |
|  | SMURF1 | -2.561 |
| **CD28 Signaling**  **in T Helper Cells**  IPA  (-log p value 2.97E00) | AKT3 | -2.566 |
|  | BCL10 | -3.117 |
|  | CD28 | -3.620 |
|  | CD247 | -3.533 |
|  | CD3E | -2.909 |
|  | CTLA4 | -2.336 |
|  | ITK | -3.185 |
|  | MALT1 | -2.134 |
|  | MAPK8 | -2.126 |
|  | PIK3C2A | -2.488 |
|  | PIK3C2B | -2.082 |
|  | PPP3CC | -2.112 |
