# Supplementary figures and images for: Critical COPD respiratory illness is linked to increased transcriptomic activity of neutrophil proteases genes
Source: BMC Res Notes. 2012 Aug 2;5:401. doi: 10.1186/1756-0500-5-401 (PMC3475085; doi:10.1186/1756-0500-5-401)

## Slide 1
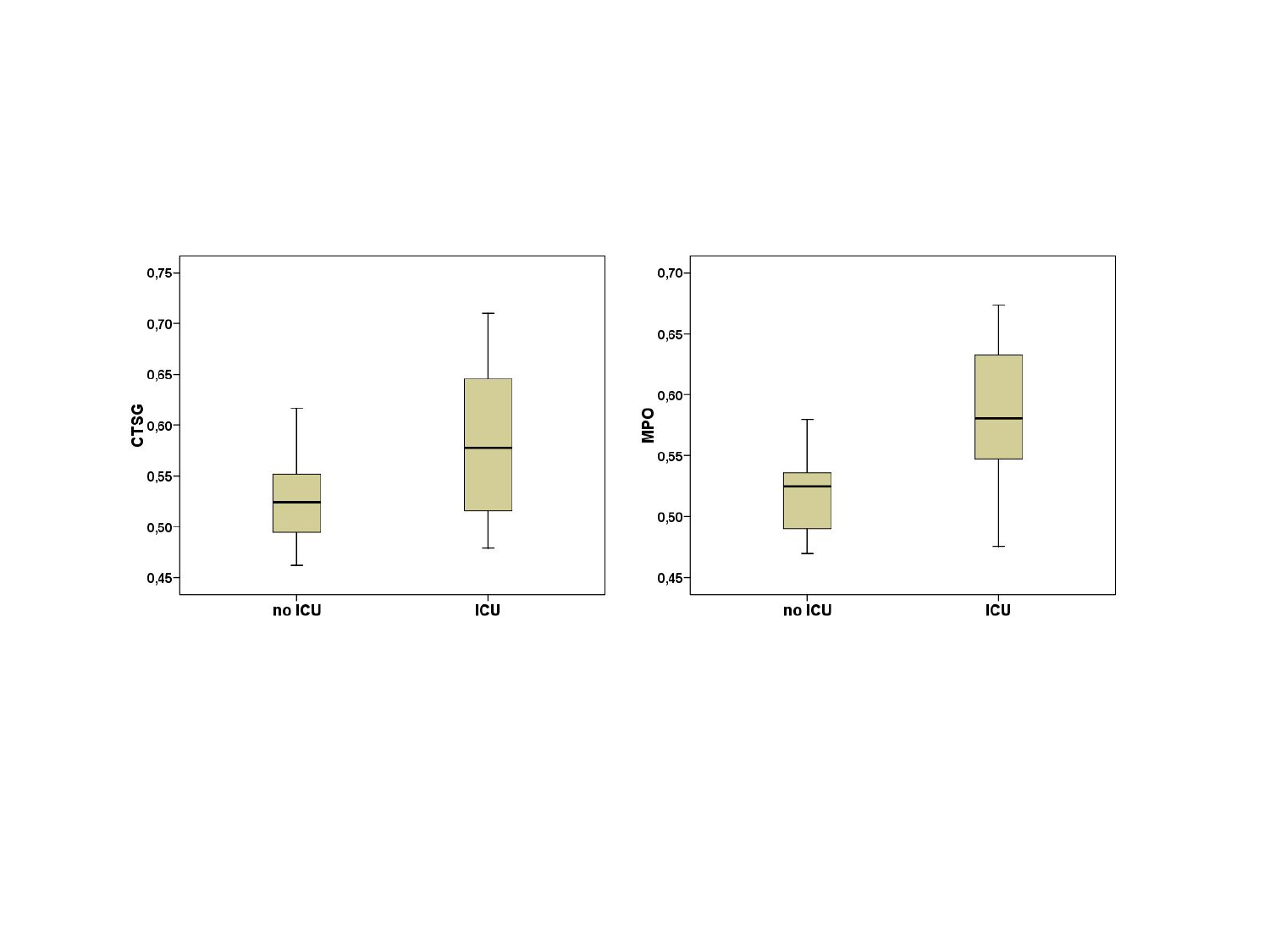

Supplement: Additional file 3 — Figure S1. Gene expression levels by Quantitative Real time PCR. Crossing points (CP) were calculated for the target gene and for the house-keeping genes (β -actin and β2-microglobulin). The ratio between the CP for the target gene / mean of the CPs for the housekeeping genes was calculated, and the inverse of this ratio is represented in each group of patients (ICU and no ICU). This way, the higher the median in the box-plot, the higher is the expression of the target gene. Differences were significant at the level p < 0.05. [file 1756-0500-5-401-S3.ppt]
